# A Study of Quantifying Thickness of Ferromagnetic Pipes Based on Remote Field Eddy Current Testing

**DOI:** 10.3390/s18092769

**Published:** 2018-08-23

**Authors:** Wei Zhang, Yibing Shi, Yanjun Li, Qingwang Luo

**Affiliations:** 1 School of Automation Engineering, University of Electronic Science and Technology of China, Chengdu 611731, China; ybshi@uestc.edu.cn (Y.S.); yjli@uestc.edu.cn (Y.L.); qwluo@std.uestc.edu.cn (Q.L.); 2 Center for Information Geoscience, University of Electronic Science and Technology of China, Chengdu 611731, China

**Keywords:** RFECT, ferromagnetic pipe, FEA, wall thickness, quantification, nonlinear fitting, MFECT

## Abstract

Remote Field Eddy Current Testing (RFECT) has broad applications in ferromagnetic pipe testing due to the same testing sensitivity to inner and outer wall defects. However, how to quantify wall thickness in the RFECT of pipes is still a big problem. According to researchers’ studies, a linear relationship exists between the wall thickness, permeability and conductivity of a pipe and the phase of the RFECT signal. Aiming to quantify wall thickness by using this linear function, it is necessary to further study the effects of pipe permeability and conductivity on the phase of the RFECT signal. When the product value of the permeability and the conductivity of a pipe remains constant, the univariate analysis and Finite Element Analysis (FEA) are employed to analyze the variations among the phase of the RFECT signal caused by different couples of permeability and conductivity. These variations are calibrated by using a nonlinear fitting method. Moreover, Multi-Frequency Eddy Current Testing (MFECT) is applied to inverse the permeability and conductivity of a pipe to compensate for the quantification analysis of wall thickness. The methods proposed in this paper are validated by analyzing the simulation signals and can improve the practicality of RFECT of ferromagnetic pipes.

## 1. Introduction

The lifeblood of exploring and transporting oil and gas is comprised of ferromagnetic pipes. Therefore, the monitoring and prevention of pipe defects are of great importance [1,2,3]. Remote Field Eddy Current Testing (RFECT) is regarded as one of the most suitable ways for detecting pipe defects because of advantages such as equal sensitivity to inner and outer defects, insensitivity to lift-off effects and contactless testing [4,5]. Recently, a lot of research has focused on overcoming the inherent drawbacks of RFECT, such as the single spectrum, long probes and weak testing signal and the achievements are remarkable [6,7,8,9,10]. In the Eddy Current Testing (ECT) of ferromagnetic pipes, including RFECT of ferromagnetic pipes, achieving wall thickness quantification is always the first priority [11,12,13,14]. Accompanied by the progress of computer and computing technology, advanced computing methods (for example, Bayesian network [15], artificial neural networks [16] and Support Vector Machine (SVM) [17]) are employed to reconstruct and quantify defects. The authors also studied and compared evaluation methods of pipe defects based on nonlinear fitting, neural networks and SVM. These methods took a long time to process the data and problems were encountered when inverting the defect size [18].

It is known that a simple linear function exists between the wall thickness, permeability and conductivity of pipe and the phase of RFECT signal [19]. This linear function can be used for the quantification analysis of wall thickness. However, this linear function is rarely used in practice because the pipe permeability varies with different magnetic flux densities and the linear function is simplified. In order to reduce the influence of pipe permeability on the ECT signal, some researchers have suggested testing ferromagnetic pipes under magnetic saturation [20,21], but the magnetizer may change the features of tested pipes. The authors concentrated on applying RFECT in practical ferromagnetic pipes testing. As such, the authors have worked out a method for removing secondary peaks in practical RFECT of ferromagnetic pipes [22] and have investigated array RFECT and pulsed RFECT of ferromagnetic pipes [23,24]. By combining the authors’ previous studies, this paper studies a method for quantifying wall thickness in RFECT of ferromagnetic pipes based on the linear function that was introduced before. In this study, the univariate analysis and the Finite Element Analysis (FEA) were employed to analyze variations among the phase of the RFECT signal caused by different couples of permeability and conductivity. These variations were calibrated by using a nonlinear fitting method. After the calibration, Multi-Frequency Eddy Current Testing (MFECT) was applied to inverse the permeability and conductivity of the pipe to compensate for the quantification analysis of wall thickness. The methods proposed in this paper were verified by analyzing simulation signals and can improve the practicality of RFECT of ferromagnetic pipes.

## 2. Methods and Models

### 2.1. Wall Thickness Analysis in RFECT of Pipes

**Figure 1:**
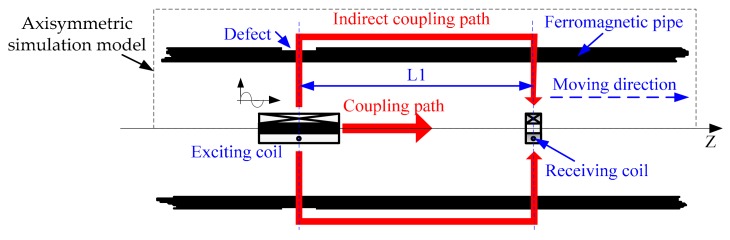
General Remote Field Eddy Current Testing (RFECT) of ferromagnetic pipes.

The general RFECT model of ferromagnetic pipes is shown in Figure 1.

As shown in Figure 1, for the general RFECT of ferromagnetic pipes, the receiving coil is set in a remote field ( the distance between the receiving coil and the exciting coil is 2.5-8 times longer than the inner diameter ) and is coaxial to the exciting coil. The exciting signal is sinusoidal, and its frequency is usually between 0 and 100 Hz. There are two transmission paths in RFECT (the coupling path and the indirect coupling path), and the inductive signal on the receiving coil mainly comes from the indirect coupling path.

In RFECT of ferromagnetic pipes, the phase of the RFECT signal can be used to estimate the pipe thickness and the relationship between the wall thickness (h), permeability (μ) and conductivity (σ) of the pipe. The phase (ϕ(radian)) of the RFECT signal [19] is shown in Equation (1).

(1) ϕ=2hΦ

where Φ is the inverse of the skin depth and is given by

(2) $$\Phi =\sqrt{\pi f\mu \sigma \;}$$

where f is the frequency of the exciting signal, and the value of f is between 0 and 100 Hz.

**Figure 2:**
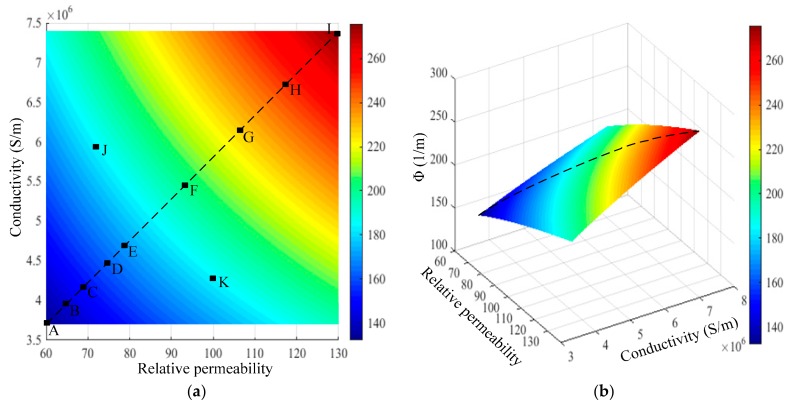
The computing results of Equation (2): (**a**) plane view and (**b**) 3D view.

According to Equations (1) and (2), the permeability and conductivity of pipe influence the phase of the RFECT signal in a product way. Generally, the relative permeability and conductivity of ferromagnetic pipes range between 60 and 130 or 3.7 and 7.4 MS/m, respectively. When the frequency of the exciting signal is 20 Hz, the computing results of Equation (2) are shown in Figure 2. As shown in Figure 2, the computing value of Φ remains constant when the product value of permeability and conductivity remains unchanged (points J and K in Figure 2 for instance). A constant value of Φ corresponds to many couples of permeability and conductivity. In order to validate the analysis above, the point E and point G in Figure 1 were chosen to be simulated using ANSYS software. In every simulation, the pipe magnetic behavior was linearly set (constant pipe permeability) and the simulation results are shown in Table 1. 

In simulations, the frequency of the exciting signal was 20 Hz, the pipe inner diameter was 157.1 × 10^−3^ m, the pipe thickness was 10.36 × 10^−3^ m, the simulation model was 2D axisymmetric, and the parameters of the exciting coil and receiving coil were set as the same as previously introduced in [22,23]. According to Table 1, when the product value of the pipe permeability and conductivity remains constant (point E), different couples of permeability and conductivity have different simulation results (such as the simulating phase in Table 1). The errors were produced when the simulating phase was used to calculate the pipe thickness directly (by using Equations (1) and (2)), and the largest relative error between the calculated pipe thickness and the pipe thickness observed in the simulation is 8.22%. In our project, the precision of relative error between a testing result and the real thickness is required to be less than 10%. The error observed for the computing method (maximum: 8.22%) is close to the maximal error requirement (10%). Therefore the phase of the RFECT signal must be calibrated before it can be used to compute the pipe thickness.

### 2.2. Calibrations of RFECT Signal

**Figure 3:**
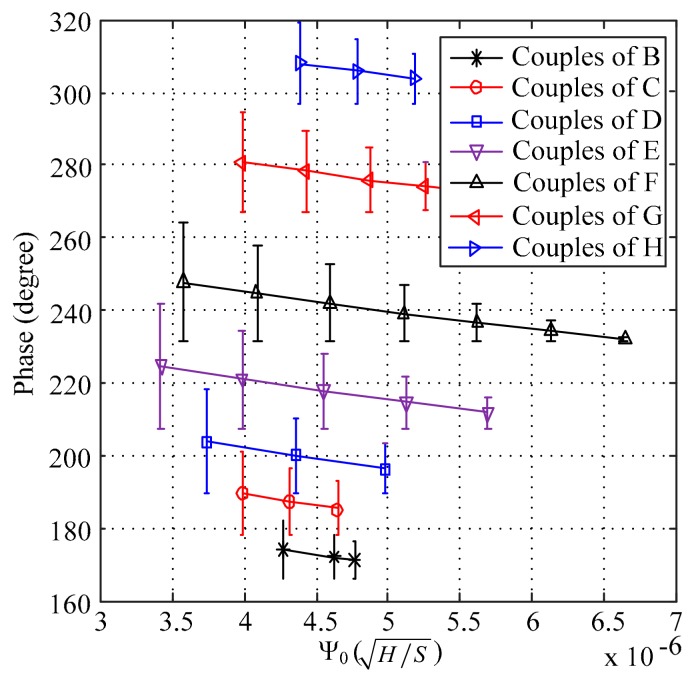
The error bars of the simulated phase when compared to the theoretical phase.

As shown in Figure 1, the value of Φ ranges between the value of point A and the value of point I, and the points (A, B, C, D, E, F, G, H and I) on the dotted line were chosen to implement the calibration of the phase of the RFECT signal. Based on the conclusionin Section 2.1, the differences between the simulated phase and the theoretical phase of the RFEC signal are shown in Figure 3.

In Figure 3, the variable of the horizontal axis is given by Equation (3)

(3) $$\Psi_{0}=\sqrt{\frac{\mu }{\sigma }\;}$$

Equation (3) was chosen because of the negative correlation between the relative permeability and the simulated phase and the positive correlation between conductivity and the simulated phase, as shown in Table 1. As shown in Figure 3, every curve demonstrates a similar trend. There was a large error between the simulated phase and the theoretical phase when the value of $\Psi_{0}$ was small, and this error becomes smaller as the value of $\Psi_{0}$ becomes bigger. 

**Figure 4:**
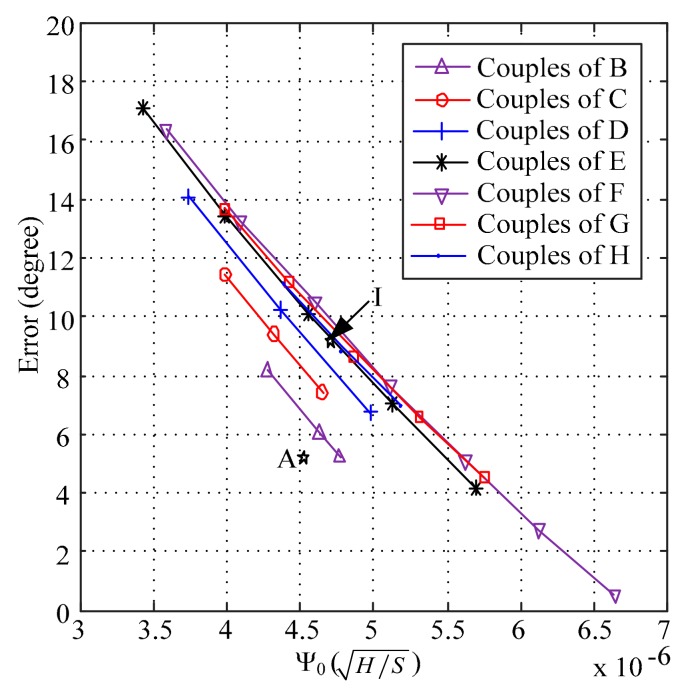
The errors between the simulated phase and the theoretical phase.

The errors in Figure 3 were extracted to implement the calibration between the simulating phase and the theoretical phase. The extracted results are shown in Figure 4. As shown in Figure 4, every curve was linear and the slopes of the curves were similar. The curves in Figure 4 were fitted by using Equation (4), and the slope of every curve is given in Table 2.

(4) $$\theta_{e}=a_{0}\Psi_{0}+b_{0}$$

where $a_{0}$ is the slope, and $b_{0}$ is the intercept.

As the slope of each curve in Figure 4 is close to each other, the average value of all slopes was used as the final slope for every linear curve in Figure 3 to simplify the calibration method. By using the average value of the slopes, and Equation (4), the intercepts of the curves in Figure 3 are given below.

**Figure 5:**
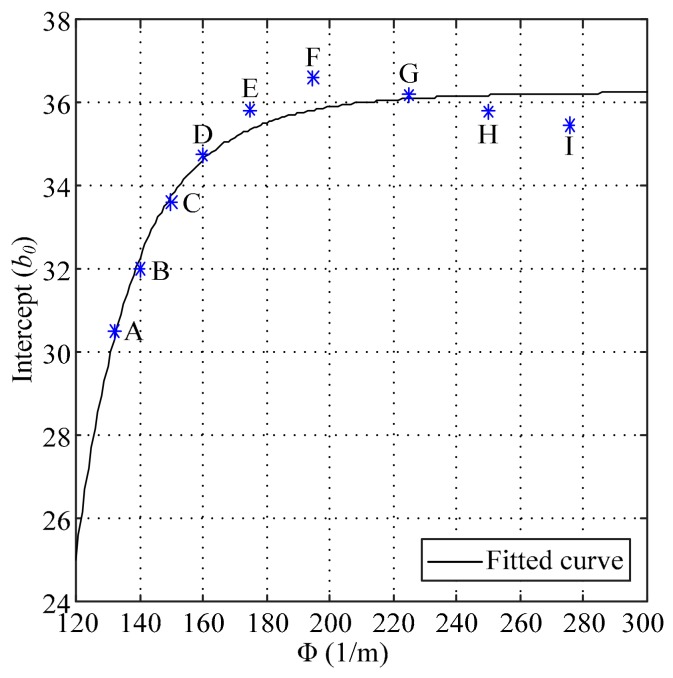
The trend of the intercepts.

According to the intercepts shown in Table 3, the factor shown in Equation (2) was applied to analyze the variation of the intercepts, and the trend is shown in Figure 5.

In Figure 5, the solid line is the fitted curve, and the function of the fitted curve is given by

(5) $$b_{0}=a_{1}\Phi^{b_{1}}+c_{1}$$

where $a_{1}$, $b_{1}$ and $c_{1}$ are the fitted coefficients, and their values are −9.706 × 10^14^, −6.70 and 36.22, respectively.

The function shown in Equation (5) was used for two reasons. The first reason was that the large constant value of $c_{1}$ makes the proportion of the deviation introduced by Φ remain small. The second reason was that Equation (5) is a simple function, and the overall fitting effect is good. 

By combining Equations (4) and (5), the calibration function was obtained as follows

(6) $$\theta_{theory}=\theta_{simulation}-\theta_{e}=\theta_{simulation}-a_{0}\Psi_{0}-a_{1}\Phi^{b_{1}}-c_{1}$$

where $\theta_{theory}$ is the theoretical phase of the RFECT signal, $\theta_{simulation}$ is the simulation phase of the RFECT signal, and the units of $\theta_{theory}$ and $\theta_{simulation}$ are degrees.

By substituting Equation (6) into Equations (1) and (2), the wall thickness of tested ferromagnetic pipes can be recomputed by

(7) $$h=\frac{\phi_{theory}}{2\Phi }=\frac{\theta_{simulation}-a_{0}\Psi_{0}-a_{1}\Phi^{b_{1}}-c_{1}}{2\Phi }\times \frac{\pi }{180}$$

where $\phi_{theory}$ is the theoretical phase in radians.

After the phase of the RFECT signal is calibrated, the computing precision of wall thickness can be improved. As analyzed, the factor $\Psi_{0}$ was used to implement the calibration. Therefore, it is necessary to study the compensation method of $\Psi_{0}$ in order to apply the calibration method in practice.

### 2.3. Compensation Method Based on MFECT

**Figure 6:**
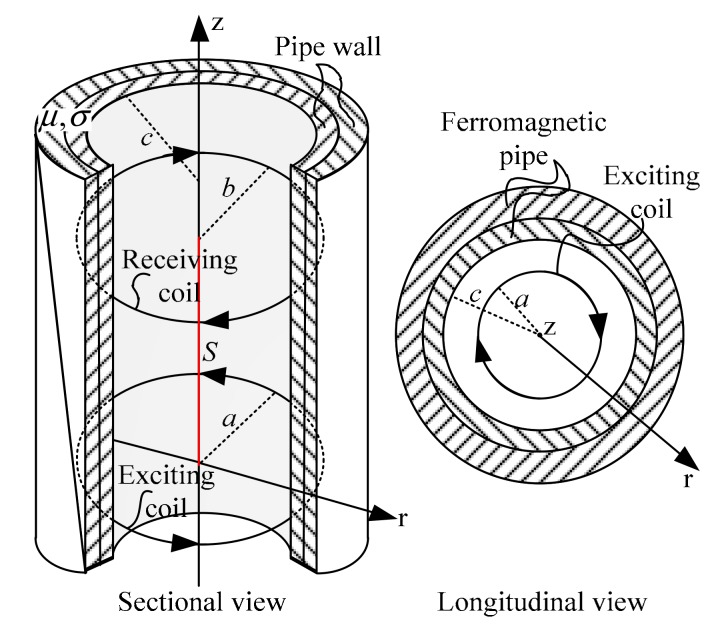
The geometric model of Multi-frequency Eddy Current Testing (MFECT) of the pipe.

As the pipe permeability is not constant at different magnetic flux densities and the analysis of pipe thickness is based on RFECT, it is better to test the permeability and conductivity of pipes under ECT conditions. When the RFECT is performed under weak magnetic induction and the testing speed of the RFECT tool is slow, the electromagnetic properties of ferromagnetic pipes are considered to be linear [25]. In our project, the testing speed of RFECT tool was as slow as 0.005 m/s and the magnitude of the RFECT signal is at millivolt level. Therefore, the permeability of ferromagnetic pipes can be regarded as constant and can be measured by using ECT which, also performs under weak magnetic flux density and low testing speeds. Thanks to the studies of Vasić and Bilas et al., a method to test the inner diameter, permeability and conductivity of pipes based on MFECT is proposed in References [26,27]. In this paper, MFECT was also used to test the permeability and conductivity of pipes and the main procedures are given concisely. According to Reference [26], the geometry used to test the permeability and conductivity of ferromagnetic pipes based on MFECT is shown in Figure 6.

In Figure 6, a is the radius of the exciting coil, b is the radius of the receiving coil (in this study, b is equal to a), c is the inner radius of the tested ferromagnetic pipe, and S is the distance between the exciting coil and the receiving coil. The exciting signals are sinusoidal, and their frequency is usually above 500 Hz. Based on Reference [26], the transfer impedance between the exciting coil and the receiving coil can be given by

(8) $$Z_{p}=Z_{0}+\Delta Z$$

where $Z_{0}$ is the transfer impedance between the exciting coil and the receiving coil when the coils are tested without pipes and ΔZ is the change in transfer impedance when the coils are tested with pipes. $Z_{0}$ and ΔZ can be described by Equations (9) and (10), respectively [27].

(9) $$Z_{0}=2j\omega \mu_{0}ab\int_{0}^{\infty }K_{1}(xa)I_{1}(xb)\;\cos(xS)dx$$

(10) $$\Delta Z=2j\omega \mu_{0}ab\int_{0}^{\infty }\Lambda I_{1}(xa)I_{1}(xb)\cos(xS)dx\;$$

where $\mu_{0}$ is the permeability of air, ω is the angular frequency of the exciting signal, $I_{1}(xb)$ and $K_{1}(xa)$ are first kind and second kind of first-order modified Bessel functions respectively. Λ is given by Equation (11)

(11) $$\Lambda =\frac{ϒxK_{0}(xc)-K_{1}(xc)\;}{ϒxI_{0}(xc)+I_{1}(xc)}$$

where

(12) $$ϒ\approx \frac{(1-j)\;}{\mu_{0}\sqrt{2\omega }}\sqrt{\frac{\mu }{\sigma }}=\frac{\left(1-j\right)}{\mu_{0}\sqrt{2\omega }}\Psi_{0}$$

Equations (11) and (12) were obtained when the skin depth is much less than the thickness of the ferromagnetic pipe [26,27]. According to Equations (8) to (12), the permeability and conductivity of pipes influence the transfer impedance between the exciting coil and the receiving coil in the way of $\Psi_{0}$. Theoretically, the value of the transfer impedance remains constant if the value of $\Psi_{0}$ remains constant. Different couples of permeability and conductivity of pipes were used to validate this feature, and the simulations were also performed using ANSYS software. The simulation results are shown in Table 4.

In simulations, the frequency of exciting signal was 600 Hz. According to Table 4, when the value of $\Psi_{0}$ remains constant, different couples of permeability and conductivity of pipes have almost no impact on the simulation results for transfer impedance (the maximal difference among the amplitudes is 0.1 V, and the maximal difference among the phases is 0.04 degree). Table 4 validates the analysis for the influence of pipe permeability and conductivity on the transfer impedance.

Moreover, not only does the permeability and conductivity of pipes influence the transfer impedance, but also the inner radius of pipes influences the transfer impedance. According to factors that influence the transfer impedance, MFECT was employed to test the inner radius, permeability and conductivity of pipes. The frequencies of the exciting signals were 600 Hz, 1500 Hz, 14,000 Hz, respectively. By exciting the three frequencies, the phase of the transfer impedances can be given by

(13) $$P=(P_{600Hz\;},P_{1500Hz},P_{14000Hz})$$

where $P_{600Hz}$, $P_{1500Hz}$ and $P_{14000Hz}$ are the phases of the transfer impedance when the frequencies of the exciting signals are 600 Hz, 1500 Hz, 14,000 Hz, respectively.

By using Finite Element Analysis (FEA), an inversion library $P\sim (c,\Psi_{0})$ was created. Based on the created inversion library, the inversion model that was used to inverse the inner radius (c), permeability (μ) and conductivity (σ) of pipes is given by

(14) $$\left(c^{*},\Psi_{0}^{*})=\arg{\min\;}_{\left(c,\Psi \right)}\{F(c,\Psi_{0})\right\}$$

where $\left(c^{*},\Psi_{0}^{*}\right)$ are the inversion results. $F(c,\Psi_{0})$ is given by

(15) $$F(c,\Psi_{0})=\frac{1}{2}\sum_{i=1\;}^{3}{\left(G(c,\Psi_{0})-T_{i}\right)}^{2}=\frac{1}{2}{\left\|e(c,\Psi_{0})\right\|}^{2}=\frac{1}{2}e{\left(c,\Psi_{0}\right)}^{T}e(c,\Psi_{0})$$

where $G(c,\Psi_{0})$ is the fitting function based on the inversion library, $T_{i}$ is the testing result of the exciting signals, and e is the error between the theoretical value and the testing result.

Aiming to implement the inversion from Equation (14) to Equation (16), the inversion method has good approximation ability and generalization ability, and the Least-Square Support Vector Regression (LSSVR) was employed to realize the inversion of the radius, permeability and conductivity of pipes. The inversions can be used to compensate for the wall thickness in the analysis of pipes, and the results are shown and discussed in the next section.

## 3. Results and Discussion

After the phase of the RFECT signal is calibrated by using the methods introduced in Section 2, the phase was used to compute the wall thickness of ferromagnetic pipes, and the results are shown in Table 5.

In Table 5, the data in the first five lines were used to implement the calibration method, and the comparisons in the first five lines were used to validate the approximation ability of the calibration method. The data in the last four lines were not involved in the calibrations, and the comparisons in the last four lines verify the generalization ability of the calibration method. According to Table 5, the biggest relative error (9.81%) between the real pipe thickness and the computing pipe thickness is close to 10% before calibration. After calibration, the biggest relative error (0.77%) between the real pipe thickness and the computing pipe thickness is below 1%. Table 5 validates the effectiveness of the calibration method in the pipe thickness computing based on RFECT.

## 4. Conclusions

This paper investigates a calibration method used to improve the computing precision of the wall thickness in the RFECT of ferromagnetic pipes. In this investigation, the differences between the phase of the RFECT signal caused by different couples of permeability and conductivity were analyzed by using univariate analysis and Finite Element Analysis (FEA). Secondly, the differences between the phase of the RFECT signal were calibrated by employing a nonlinear fitting method. Finally, aiming to compensate the calibration of differences among the phase of the RFECT signal, the method used to inverse the permeability and conductivity of pipes was studied based on Multi-frequency Eddy Current Testing (MFECT). The proposed method was analyzed based on the linear performance of the magnetization curve (B-H curve) of ferromagnetic pipes, and RFECT and MFECT were both required to be performed under a low testing speed and a weak magnetic flux density. Due to the fact that appropriate pipes (pipes with different permeability and conductivity, but with constant product value of permeability and conductivity) that can be used to implement the validation are difficult to obtain, the methods studied in this paper were validated by simulation, using ANSYS software, and future work is focused on applying the proposed methods in practice by combining theoretical analysis and general pipe experiments. The methods proposed in this paper can be used to improve the practicality of the RFECT of ferromagnetic pipes.

## Figures and Tables

**Table 1:** Influence of different couples of permeability and conductivity on pipe thickness analysis.

**Point E (Φ = 175 (1/m))**		**ANSYS Simulation**		**Calculated Results**	
**Relative Permeability**	**Conductivity (MS/m)**	**Pipe Thickness (m × 10^−3^)**	**Simulated Phase (degree)**	**Pipe Thickness (m × 10^−3^)**	**Relative Error (%)**
60	6.465	10.36	224.85	11.21	8.22
70	5.541	10.36	221.21	11.03	6.48
80	4.848	10.36	217.85	10.86	4.83
90	4.310	10.36	214.78	10.71	3.38
100	3.879	10.36	211.93	10.57	2.01
**Point G (Φ = 225 (1/m))**		**ANSYS Simulation**		**Calculated Results**	
**Relative Permeability**	**Conductivity (MS/m)**	**Pipe Thickness (m × 10^−3^)**	**Simulated Phase (degree)**	**Pipe Thickness (m × 10^−3^)**	**Relative Error (%)**
90	7.124	10.36	280.79	10.89	5.12
100	6.412	10.36	278.29	10.79	4.18
110	5.829	10.36	275.93	10.70	3.30
120	5.343	10.36	273.70	10.62	2.47
130	4.932	10.36	271.60	10.53	1.68

**Table 2:** The fitted slope for each curve.

Point	B	C	D	E	F	G	H	Average
**Slope** ($a_{0}$ × 10^6^)	−5.876	−6.038	−5.935	−5.670	−5.161	−5.188	−5.208	−5.582

**Table 3:** The fitted intercept for each curve.

Point	A	B	C	D	E	F	G	H	I
**Intercept** ($b_{0}$)	30.39	31.90	33.52	34.68	35.79	36.56	36.14	35.72	35.42

**Table 4:** Influence of different couples of permeability and conductivity on the transfer impedance.

**Point M (Ψ_0_ = 4.244 × 10^−6^ (** $\sqrt{H/S}$ **))**		**Transfer Impedance**	
**Relative Permeability**	**Conductivity (MS/m)**	**Amplitude (V)**	**Phase (degree)**
60	4.187	6.59	15.26
70	4.885	6.58	15.25
80	5.582	6.58	15.24
90	6.280	6.58	15.23
100	6.978	6.58	15.22
**Point N (Ψ _0_ = 5.401 × 10^−6^ (** $\sqrt{H/S}$ **))**		**Transfer Impedance**	
**Relative Permeability**	**Conductivity (MS/m)**	**Amplitude (V)**	**Phase (degree)**
90	3.877	7.05	12.62
100	4.308	7.05	12.62
110	4.739	7.04	12.61
120	5.169	7.04	12.61
130	5.600	7.04	12.61

**Table 5:** Comparison of the computing results of wall thickness.

Relative Permeability	Conductivity (MS/m)	Real pipe Thickness (m × 10^−3^)	Before Calibration		After Calibration	
			Pipe Thickness (m × 10^−3^)	Relative Error (%)	Pipe Thickness (m × 10^−3^)	Relative Error (%)
60	6.465	10.36	11.21	8.22	10.40	0.39
70	6.880	10.36	11.09	7.05	10.38	0.19
90	7.124	10.36	10.89	5.12	10.35	0.10
110	7.196	10.36	10.75	3.76	10.34	0.19
130	7.400	10.36	10.65	2.80	10.34	0.19
60	7.400	10.36	11.24	8.49	10.41	0.48
130	3.700	10.36	10.38	0.19	10.44	0.77
60	7.400	8.36	9.18	9.81	8.35	0.12
100	5.550	12.36	12.72	2.91	12.33	0.24
